# Profile of the cohort of people being treated for HIV infection in the SUS, Brazil, 2015–2018

**DOI:** 10.11606/s1518-8787.2023057005256

**Published:** 2023-09-14

**Authors:** Ana Paula Sayuri Sato, Maria Ines Battistella Nemes, Ana Maroso Alves, Evelyn Lima de Souza, Barbara dos Reis Santos, Luceime Olivia Nunes, Angélica Carreira dos Santos, Aline Kumow, Felipe Parra do Nascimento

**Affiliations:** I Universidade de São Paulo Faculdade de Saúde Pública Departamento de Epidemiologia São Paulo SP Brazil Universidade de São Paulo. Faculdade de Saúde Pública. Departamento de Epidemiologia. São Paulo, SP, Brazil; II Universidade de São Paulo Faculdade de Medicina Departamento de Medicina Preventiva São Paulo SP Brazil Universidade de São Paulo. Faculdade de Medicina. Departamento de Medicina Preventiva. São Paulo, SP, Brazil; III Universidade de São Paulo Faculdade de Saúde Pública Programa de Pós-Graduação em Saúde Pública São Paulo SP Brasil Universidade de São Paulo. Faculdade de Saúde Pública. Programa de Pós-Graduação em Saúde Pública. São Paulo, SP, Brasil; IV Universidade de São Paulo Faculdade de Medicina Departamento de Pediatria São Paulo SP Brazil Universidade de São Paulo. Faculdade de Medicina. Departamento de Pediatria. São Paulo, SP, Brazil

**Keywords:** HIV, Cohort Studies, Brazilian Unified Health System, Health Services Research, Tuberculosis

## Abstract

**OBJECTIVE:**

To build an integrated database of individual and service data from the cohort of people who started antiretroviral therapy (ART), from 2015 to 2018, in Brazil.

**METHODS:**

Open cohort study that includes people aged 15 years or older who started ART from 2015 to 2018, with follow-up in services of the Brazilian Unified Health System (SUS), and who responded to the 2016/2017 Qualiaids national survey. The source of individual data was the related HIV database, derived from the probabilistic linkage between data from the SUS systems of diagnostic information, medication, tests, and deaths. The data source for the services was the services’ response database to the Qualiaids survey. After analysis of consistency and exclusions, the database of individuals was deterministically related to the database of services.

**RESULTS:**

The cohort comprised 132,540 people monitored in 941 SUS services. Of these services, 59% are located in the Southeast region and 49% followed 51 to 500 cohort participants. The average performance of organization and management of patient care ranged from 29% to 75%. Most of the cohort participants are male, black and mixed, aged between 20 and 39 years old, and have between 4 and 11 years of schooling. Median baseline T-CD4 was 419 cells/mm^3^, 6% had an episode of tuberculosis, and 2% died of HIV disease.

**CONCLUSION:**

For the first time in Brazil, this cohort provides the opportunity for a joint analysis of individual factors and services in the production of positive and negative clinical outcomes of HIV treatment.

## INTRODUCTION

In Brazil, since the implementation of free and universal access to combined antiretroviral therapy (ART) in 1996, the treatment of HIV infection has achieved positive results in the national rates of viral suppression^1^ and survival, mainly after the incorporation of potent antiretroviral drugs in the 2010s^2^. Outpatient services of the Brazilian Unified Health System (SUS) are the exclusive distributors of antiretroviral drugs in the country. All persons must be registered with a SUS service to receive the drugs, regardless of whether the prescription originates from a SUS service or from the private sector.

Despite the easy access to medication and medical treatment assistance, significant regional and social differences in viral suppression^3^and survival^4^persist, reflecting the country’s social and racial inequality^5^. Additionally, heterogeneity in the organization of care provided by treatment services^6^may contribute to the observed differences^7,8^.

The SUS maintains national systems for continuous registration of case and death notifications. Specifically for HIV, there is a system for continuous registration of antiretroviral dispensing for all people on ART in the country. For those undergoing follow-up at SUS services, a system continuously records T-CD4 lymphocyte (CD4) and viral load (VL) counts. However, there is no single key that makes it possible to identify the records of the same individual in different databases. Consequently, analyses of the information set depend on linkage techniques. Information on the services comes from periodic national surveys on the local organization of care in outpatient HIV treatment services of the SUS (Qualiaids questionnaire)^9,10^.

The Qualiaids Brazil Cohort, conceived from the linkage between these databases, provides an opportunity for an integrated analysis of the role of the characteristics of people and treatment services in the outcomes of HIV treatment across the country. This article describes the construction process and the structure of the cohort, in addition to presenting the main characteristics of the participants and SUS health services in the treatment of HIV.

## METHODS

The Qualiaids Brazil Cohort is an open cohort that included people aged 15 years or older who started ART between 2015 and 2018, whose treatment was monitored at SUS outpatient services, and who responded to the 2016/2017 Qualiaids national survey.

The beginning of treatment was defined as the registered date of the first dispensing of ART drugs, while the end of follow-up was the date of death due to HIV disease or loss of follow-up (a person who, after 100 days without dispensing, had no other record until December 31, 2018, or record of death due to HIV disease) or administrative censure on December 31, 2018.

The inclusion of people aged 15 years or older was because the clinical treatment protocol does not distinguish between adolescents and adults^11^. The follow-up period considered the applicability of the results of the national services survey, carried out from 2016 to 2017^10^.

### Data Source of Individuals

In Brazil, the Ministry of Health (MS) consolidates the records of health care services for HIV infection in the following information systems: *Sistema de Informação de Agravos de Notificação* (Information System for Notifiable Diseases - Sinan), to notify new cases; *Sistema de Informação sobre Mortalidade* (Mortality Information System - SIM), for deaths; *Sistema de Controle Logístico de Medicamentos* (Medication Logistic Control System - Siclom), for ART dispensations for all people on ART residing in the country; and *Sistema de Controle de Exames Laboratoriais* (System for the Control of Laboratory Tests - Siscel), T-CD4 lymphocyte count tests, and VL tests for people being followed up at SUS services.

Since these systems do not have a unique identifying field, MS lists them probabilistically using the Reclink^12^program, validated for HIV systems^13,14^. The related database constitutes the basis for the health indicators of the national epidemiological bulletins^15,16^and has already been used in longitudinal studies of national scope^1,2,4^.

The database selection for the period from 2015 to 2018 with anonymized data and unique identifier, obtained in 2020, was the primary source of data on individuals. The complete original databases of Siclom and Siscel were also accessed to identify inconsistencies.

The consistency analysis aimed to identify possible errors in the probabilistic linkage (probably different people who were paired by the program and had the original records gathered in a single identifying code) and possible errors in the original record when filling out the information systems by the service. At this stage, a deterministic linkage was performed with the Siclom and Siscel databases to confirm the true pairs, and the distributions of the clinical variables (dispensing of medication and T-CD4 and VL tests) were verified and cross-tabulations were made to identify errors.

After excluding inconsistencies, the individual’s treatment follow-up health system was defined: whether in SUS outpatient services or in private services. People with: i) two or more VL test results; or ii) only one VL test result and loss to follow-up due to noncompliance or death within 66 days after the first ART dispensing were defined as being followed up at the SUS. This interval was based on the clinical protocol for ART in adults, which recommends performing a VL exam within 56 days after starting ART^11^. Since this is a “real life” study, ten days were added to the recommended interval, allowing for delays arising from the service and/or the user.

The follow-up service was defined as the one that requests VL exams. For people with only one service requesting the VL test, the SUS service attribution was direct; for those with more than one VL requesting service, the assigned service was the one in which there were more VL requests or in which the follow-up was longer.

**Figure 1:**
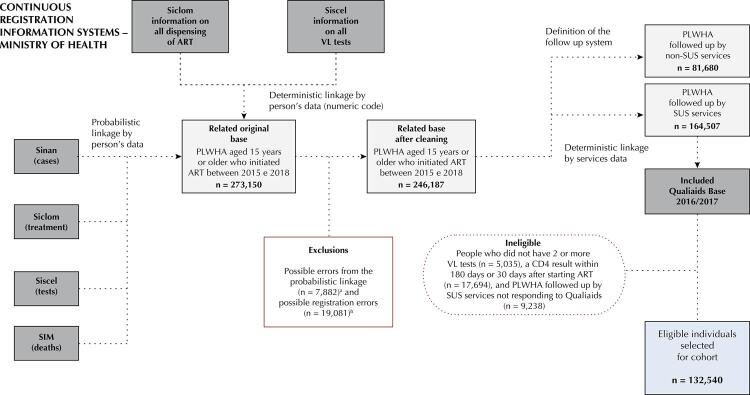
Flowchart of the construction process of the Qualiaids Brazil Cohort. Brazil, 2022.

Then, individuals with at least two VL tests and a record of the first CD4 test collected within 180 days before and 30 days after starting ART were selected. This time interval was based on the great variation of intervals observed in the original database, frequent in studies that use surveillance data^17^. The procedure resulted in a database of sociodemographic and clinical characteristics of individuals (Figure 1).

Sinan: *Sistema de Informação de Agravos de Notificação* (Informatiom System for Notifiable Diseases); Siclom: *Sistema de Controle Logístico de Medicamentos* (Medication Logistic Control System) ; Siscel: *Sistema de Controle de Exames Laboratoriais de CD4+/CD8+ e Carga Viral do HIV* (CD4+/CD8+ and HIV Viral Load Laboratory Test Control System); SIM: *Sistema de Informação sobre Mortalidade* (Mortality Information System); ART: antiretroviral therapy; PLWHA: people living with HIV/aids; VL: viral load.

^a^ Incompatibility of Sinan data (n = 593), dispensing after death (n = 300), deaths prior to treatment initiation (n = 649), notification of tuberculosis after death (n = 67), duplicate CD4 test results (n = 2), duplicate VL test results (n = 261), duplicate dispensing records (n = 257), incorrect registration of ART initiation (5,073), interruption of ART and subsequent VL result undetectable (n = 160).

^b^ Record of first undetectable VL result (n = 19,081).

### Services Data Source

The primary base of responses to the 2016/2017 Qualiaids national survey was used, answered by the technical team of the SUS outpatient services. The Qualiaids questionnaire is a validated and periodically updated assessment instrument, already answered in 2002, 2007, and 2010^6,18^. It contains questions about general characteristics of the service (type, number of people on ART, and infrastructure) and 60 questions that assess performance, grouped in the domains of access and readiness, medical care, multidisciplinary care, management and monitoring of the quality of care provided, and communication with users, local population, civil society institutions, and local health network. The percentage of positive responses to the evaluative questions yields the service’s overall performance score.

### Linkage Between the Database of Individuals and Services

The individual and service databases were linked in a deterministic way through the service location data, present in both (Figure 1).

The final database of the Qualiaids Brazil Cohort contains the individuals’ sociodemographic and clinical variables, and the programmatic variables of the health services that follow them up (Box).

**Box:** Characteristics of individuals and services included in the Qualiaids Brazil Cohort. Brazil, 2022.

Characteristic	Categories/units	Base
**Individual**		
Date of birth	day:month:year	Related base
Sex	male; female; ignored	Related base
Race/skin color	white; black; yellow; mixed; indigenous; ignored	Related base
Education, years of schooling	no schooling; 1–3; 4–7; 8–11; ≥12; ignored	Related base
Exposure category	homosexual; heterosexual; bisexual; injecting drug users; hemophiliac; transfusion; biological material accident; vertical transmission; ignored	Related base
Date of tuberculosis diagnosis	day:month:year	Related base
Closing date of the tuberculosis case	day:month:year	Related base
CD4 collection dates	day:month:year	Siscel
CD4 results (LT-CD4 cell count)	mm^3^	Siscel
Date of registration of the first dispensing	day:month:year	Related base
Dates of ARV delivery	day:month:year	Siclom
Dates of viral load collections	day:month:year	Siscel
First viral load result	undetectable; detectable or above detection limit	Siscel
**Service**		
Metropolitan Region (MR) of the service	Brazilian MR	Qualiaids Survey
Follow-up service type	outpatient; Primary Health Care	Qualiaids Survey
Macro-region of Brazil where the service is located	N; NE; SE; S; MW	Qualiaids Survey
Service located in the capital	yes; no	Qualiaids Survey
Number of people who started ART under follow-up at the service	numeric	Siscel
Overall score on the Qualiaids questionnaire	numeric	Qualiaids Survey
Domain score: access and Readiness	numeric	Qualiaids Survey
Score in the subdomain: medical care	numeric	Qualiaids Survey
Score in the subdomain: multidisciplinary care	numeric	Qualiaids Survey
Score in the subdomain: management and monitoring of the quality of care provided	numeric	Qualiaids Survey
Score in the subdomain: communication with users, local population, local health network, civil society institutions	numeric	Qualiaids Survey
Service infrastructure	sufficient; acceptable; insufficient	Qualiaids Survey

Siclom: *Sistema de Controle Logístico de Medicamentos* (Medication Logistic Control System); Siscel: *Sistema de Controle de Exames Laboratoriais de CD4+/CD8+ e Carga Viral do HIV* (CD4+/CD8+ and HIV Viral Load Laboratory Test Control System); ART: antiretroviral therapy; ARV: antiretroviral; N: North; NE: Northeast; SE: Southeast; S: South; MW: Midwest.

### Statistical Analysis

Individual variables were described according to absolute and relative frequencies. The frequencies of the service’s general characteristics were presented according to the Brazilian macro-regions, while the service performance scores appear according to domain and macro-region.

### Ethical Considerations

The project was approved by the Human Research Ethics Committee of the Faculdade de Medicina da Universidade de São Paulo (USP) on January 23, 2020 (CAAE 27659220.3.0000.0065; Opinion 3.807.435).

## RESULTS

The Qualiaids Brazil Cohort included 132,540 people living with HIV/AIDS (PLWHA), considering the eligibility criteria. Linking SUS secondary databases resulted in 273,150 observations, of which 7,882 (2.9%) were classified as inconsistent and excluded. A total of 19,081 (7%) records of people with a first undetectable viral load were also identified and excluded (Figure 1).

Among the cohort participants, 69.7% (92,361) were male, 50.6% (67,004) black and mixed, 64.3% (85,206) were aged between 20 and 39 years, and 54.8% (72,642) had 4 to 11 years of schooling. The transmission category was heterosexual for 36% (47,657) but ignored for 34.1% (45,257) of participants. Median baseline CD4 was 419 cells/mm^3^. A total of 25.9% (34,398) of participants started treatment with CD4 counts less than 200 cells/mm^3^, and 5.8% (7,747 individuals) had at least one episode of active tuberculosis and 1.9% (2,515) died of HIV disease during the study period (Table 1).

**Table 1:** Profile of individuals included in the Qualiaids Brazil Cohort. Brazil, 2022 (n = 132,570).

Characteristic	n	%
Sex		
Male	92,361	69.7
Female	40,177	30.3
Ignored	2	0
Race/Color		
White	54,625	41.2
Black and mixed	67,004	50.6
Yellow	921	0.7
Indigenous	258	0.2
Ignored	9,732	7.3
Age (in years)		
15–19	5,962	4.5
20–39	85,206	64.3
40–49	23,961	18.1
≥ 50 years	17,411	13.1
Education (years of schooling)		
0–3	11,024	8.3
4–11	72,642	54.8
≥ 12 years	24,251	18.3
Ignored	24,623	18.6
Exposure category		
Homosexual/bisexual	37,397	28.2
Heterosexual	47,657	36.0
Intravenous drug users	1,539	1.2
Others	690	0.5
Ignored	45,257	34.1
Active tuberculosis registry		
Yes	7,747	5.8
Death from HIV disease		
Yes	2,515	1.9
Year of initiation of HIV treatment		
2015	36,979	27.9
2016	36,649	27.7
2017	36,103	27.2
2018	22,809	17.2
Initial count of T-CD4 lymphocytes (cells/mm^3^)		
< 200	34,398	25.9
200–349	25,718	19.4
350–499	27,140	20.5
> 499	45,284	34.2

**Figure 2:**
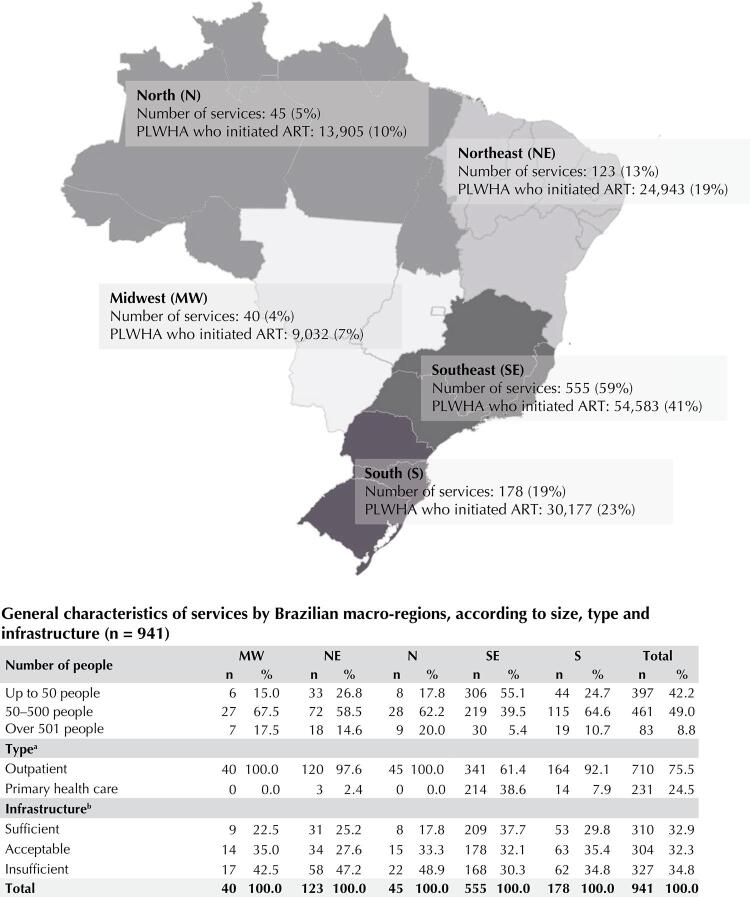
SUS HIV treatment services and number of people included in the cohort who started ART between 2015 and 2018 according to the country’s macro-region. Qualiaids Brazil cohort. Brazil, 2022. (n = 941).

ART: antiretroviral therapy; PLWHA: people living with HIV/aids.

^a^ Services were classified based on the response to the question about the type of service in the Qualiaids questionnaire. “Outpatient type” for the answers: exclusive outpatient clinic for specialized care for patients with HIV/AIDS, STD, and viral hepatitis outpatient clinic; outpatient clinic specializing in infectious diseases; specialized assistance team included in a primary care service; outpatient care for various specialties and hospital outpatient clinic; “Type of primary care” for the answers: basic health unit, health center, family health.

^b^ The services’ infrastructure was calculated based on the answers to 45 questions in the Qualiaids questionnaire on the availability of: initial assessment and follow-up tests, complementary tests for evaluation of additional clinical conditions common in HIV/AIDS patients, referrals to specialist physicians, assistance modalities and other health professionals, and availability of medicines and supplies for prevention and diagnosis. Services with up to 49% of the expected infrastructure were classified as “insufficient,” those with infrastructure between 50% and 64% were defined as “acceptable” and those with more than 65% of available infrastructure were classified as “sufficient.”

The Qualiaids Brazil Cohort comprised 941 SUS health services, of which 59% (555) are located in the Southeast region, 49% (461) concentrated from 51 to 500 participants, 75.5% (710) were characterized as “outpatient type”, and 34.8% (327) had infrastructure classified as insufficient (Figure 2).

The average performance of the care organization ranged from 29% to 75% of the expected standard. The domain of management and monitoring of the quality of care provided had the worst score (Table 2).

**Table 2:** Performance of SUS outpatient services that accompany people who started HIV treatment between 2015 and 2018 according to the mean proportion of positive responses to the Qualiaids 2016/2017 survey. Qualiaids Brazil cohort. Brazil, 2022. (n = 941).

Domains rated^a^	Midwest			Northeast			North			Southeast			South			Brazil		
					
	score	mean	SD	score	mean	SD	score	mean	SD	score	mean	SD	score	mean	SD	score	mean	SD
Access and readiness	0.53	0.5	0.2	0.5	0.5	0.21	0.55	0.5	0.18	0.61	0.63	0.23	0.53	0.5	0.2	0.58	0.63	0.22
Medical care	0.56	0.6	0.17	0.57	0.6	0.21	0.56	0.6	0.2	0.67	0.7	0.21	0.6	0.6	0.19	0.63	0.6	0.21
Multidisciplinary care	0.76	0.79	0.19	0.72	0.71	0.26	0.76	0.86	0.23	0.76	0.86	0.24	0.71	0.79	0.26	0.75	0.86	0.24
Management and monitoring of the quality of care provided	0.33	0.3	0.2	0.34	0.3	0.16	0.29	0.3	0.18	0.35	0.3	0.17	0.32	0.3	0.16	0.34	0.3	0.17
Communication with users, local population, local health network, civil society institutions	0.22	0.14	0.16	0.26	0.29	0.19	0.26	0.29	0.19	0.31	0.29	0.2	0.26	0.29	0.19	0.29	0.29	0.2
Total	0.5	0.48	0.1	0.49	0.52	0.15	0.5	0.5	0.13	0.57	0.58	0.15	0.51	0.52	0.13	0.54	0.55	0.15

SD: standard deviation.

^a^ The 60 evaluation questions of the Qualiaids questionnaire are grouped into five domains, with the following distribution: 16 questions about activities carried out in the first appointment, availability of HIV testing, pre- and post-exposure prophylaxis and types of care provided by the service make up the “access and readiness” domain; 20 questions about the activities performed by the physician make up the “medical care” domain; 7 questions about the activities carried out by nurses, pharmacists, psychologists, social workers and nursing technicians constitute the “multiprofessional care” domain; 10 questions about holding meetings, monitoring and records form the domain of “management and monitoring of the quality of care provided”; and 8 questions about types and forms of service communication make up the domain “communication with users, local population, local health network, civil society institutions”.

## DISCUSSION

It was possible to build a cohort that, in an unprecedented way in Brazil, integrates available but previously unrelated data on people undergoing HIV treatment and the SUS health services that assist them.

Data from administrative databases have been used in several studies on HIV^21^, but require detailed consistency analysis, even more crucial in a study like this one, which combines data from a survey with those from a previously related database. Information on sociodemographic and clinical characteristics comes from the probabilistic linkage conducted annually by the Ministry of Health, used in studies published in journals with a selective peer review policy^1,2,4^. However, additional assessments of potential registration or unique identifying code assignment errors were performed against the original major bases.

There were 19,081 (7%) records of people with a first undetectable VL result. Of these, 520 records were identified in the VL history that seem to be from people who maintain undetectable VL for a long time (“elite controllers”), whose estimated prevalence is less than 1%^1,24^. So, it is likely that the majority of cases result from so-called “delayed registration” in the Siclom, that is, people who started treatment, received medication, but were not immediately registered in the system. Errors like this reflect unevenness in the quality of records across services. The errors whose most likely origin was the probabilistic linkage occurred in a smaller proportion (2.7%). All errors were subject to a detailed report to the federal HIV management of the MS, responsible for managing the systems and executing the linkage. Finally, we emphasize that the linkage of systems at the national level, through a unique identifying code, would be the ideal scenario. While this condition does not exist, probabilistic linkages remain valuable sources for real-life studies^23^.

The inclusion only of people who undergo clinical and laboratory follow-up in the SUS — a requirement dictated by the study design—implied the exclusion of 30% of individuals. Among them, 63% are people who use SUS services only to dispense medications prescribed by private services, thus, they are not obliged to provide clinical information to the SUS information systems. This exclusion does not represent a limitation since it stems from an inherent characteristic of the organization of health care in Brazil. It represents, on the contrary, a strength, since this is the first Brazilian national study that adjusts inclusion according to this characteristic. Additionally, the unprecedented estimate of the relative role of the public and private systems in HIV care in Brazil was part of a previously published product of this study^25^.

Among individuals monitored by SUS services, the absence of VL and/or CD4 records implied exclusion of 8%. Although timely laboratory monitoring is recommended in the clinical treatment protocol^11^, its performance depends, above all, on the correct observance by the attending physician. The inadequacy of laboratory monitoring has already been pointed out in Brazil^26^ and Latin America^27^. The non-response of the services to the survey excluded only 3%.

The participants’ sociodemographic and clinical profile is similar to that observed in a previous national cohort^1,2^. The service profile confirms the structural and performance heterogeneity pointed out in previous studies^6^. It highlights the insufficiency of quality management and monitoring, which particularly affects the identification and intervention of people who fail treatment and/or abandon treatment^28,29^ and plays a crucial role in improving the overall performance of the care service^30^.

The linkage between individual and service databases makes it possible, for the first time in the country, to establish a dialogue with international studies of a similar design^8,33^. It is therefore expected that the Qualiaids Brazil Cohort will contribute to the construction of evidence in the area, based on analyses that integrate the different levels involved in determining the results of the HIV treatment of people monitored in the SUS services.
