# Supplementary material for: Profile of the cohort of people being treated for HIV infection in the SUS, Brazil, 2015–2018
Source: Rev Saude Publica. 2023 Sep 14;57:66. doi: 10.11606/s1518-8787.2023057005256 (PMC10519680; doi:10.11606/s1518-8787.2023057005256)
Supplement: Translation

# Translation: Perfil da coorte de pessoas em tratamento da infecção por HIV no SUS, Brasil, 2015–2018

**Authors:** Ana Paula Sayuri Sato, Maria Ines Battistella Nemes, Ana Maroso Alves, Evelyn Lima de Souza, Barbara dos Reis Santos, Luceime Olivia Nunes, Angélica Carreira dos Santos, Aline Kumow, Felipe Parra do Nascimento

**Affiliations:** I Brazil Universidade de São Paulo. Faculdade de Saúde Pública. Departamento de Epidemiologia. São Paulo, SP, Brazil; II Brazil Universidade de São Paulo. Faculdade de Medicina. Departamento de Medicina Preventiva. São Paulo, SP, Brazil; III Brasil Universidade de São Paulo. Faculdade de Saúde Pública. Programa de Pós-Graduação em Saúde Pública. São Paulo, SP, Brasil; IV Brazil Universidade de São Paulo. Faculdade de Medicina. Departamento de Pediatria. São Paulo, SP, Brazil

**Keywords:** HIV, Estudos de Coortes, Sistema Único de Saúde, Pesquisa sobre Serviços de Saúde, Tuberculose

## Abstract

**OBJETIVO:**

Construir uma base integrada de dados individuais e dos serviços da coorte de pessoas que iniciaram terapia antirretroviral (TARV) entre 2015 e 2018 no Brasil.

**MÉTODOS:**

Estudo de coorte aberta que incluiu pessoas de 15 anos ou mais que iniciaram TARV entre 2015 e 2018, com acompanhamento em serviços do Sistema Único de Saúde (SUS), e que responderam ao inquérito nacional Qualiaids de 2016/2017. A fonte de dados individuais foi o banco relacionado do HIV, proveniente do relacionamento probabilístico entre dados dos sistemas de informação de diagnóstico, medicação, exames e óbitos do SUS. A fonte de dados dos serviços foi o banco de respostas dos serviços ao inquérito Qualiaids. Após análise de consistência e exclusões, o banco dos indivíduos foi relacionado deterministicamente com o banco de serviços.

**RESULTADOS:**

A coorte reuniu 132.540 pessoas acompanhadas em 941 serviços do SUS. Desses serviços, 59% localizam-se na região Sudeste e 49% acompanharam 51 a 500 participantes da coorte. O desempenho médio de organização e gerência da assistência ao paciente variou de 29% a 75%. A maioria dos participantes da coorte é do sexo masculino, preto e pardo, com idade entre 20 e 39 anos e tem entre 4 e 11 anos de escolaridade. O T-CD4 mediano basal foi de 419 células/mm^3^, 6% tiveram episódio de tuberculose e 2% foram a óbito por doença do HIV.

**CONCLUSÃO:**

A coorte oportuniza pela primeira vez no Brasil a análise conjunta de fatores individuais e dos serviços na produção dos desfechos clínicos positivos e negativos do tratamento do HIV.

## INTRODUÇÃO

No Brasil, desde a implantação do acesso livre e universal à terapia antirretroviral combinada (TARV) em 1996, o tratamento da infecção pelo HIV vem obtendo resultados positivos nas taxas nacionais de supressão viral^1^ e de sobrevida, principalmente a partir da incorporação dos potentes antirretrovirais na década de 2010^2^. Os serviços ambulatoriais do Sistema Único de Saúde (SUS) são os distribuidores exclusivos de medicamentos antirretrovirais no país. Todas as pessoas devem ser registradas em um serviço do SUS para receber os medicamentos, independentemente de a prescrição originar-se de um serviço do SUS ou do setor privado.

Em que pese o acesso facilitado à medicação e à assistência médica do tratamento, persistem importantes variações regionais e sociais de supressão viral^3^ e sobrevida^4^ que espelham a desigualdade social e racial do país^5^. Adicionalmente, a heterogeneidade na organização do cuidado prestado pelos serviços de tratamento^6^ pode contribuir para as variações observadas^7,8^

O SUS mantém sistemas nacionais de registro contínuo de notificação de casos e de óbitos. Especificamente para o HIV, há sistema de registro contínuo das dispensações de antirretrovirais para todas as pessoas em TARV do país. Para aquelas em seguimento nos serviços do SUS, um sistema registra continuamente os exames de contagem de linfócitos T-CD4 (CD4) e de carga viral (CV). Porém, não há uma chave única que possibilite identificar os registros de um mesmo indivíduo nos diferentes bancos de dados. Como consequência, análises do conjunto de informações dependem de técnicas de relacionamento. As informações sobre os serviços são provenientes de inquéritos nacionais periódicos sobre a organização local da assistência nos serviços ambulatoriais de tratamento do HIV do SUS (questionário Qualiaids)^9,10^.

A Coorte Qualiaids Brasil, concebida a partir do relacionamento entre esses bancos, oportuniza a análise integrada do papel das características das pessoas e dos serviços de tratamento nos desfechos do tratamento do HIV em todo o país. Este artigo descreve o processo de construção e a estrutura da coorte, além de apresentar as principais características dos participantes e dos serviços de saúde do SUS no tratamento do HIV.

## MÉTODOS

A Coorte Qualiaids Brasil é uma coorte aberta que incluiu pessoas de 15 anos ou mais que iniciaram TARV entre 2015 e 2018, cujo tratamento foi acompanhado em serviços ambulatoriais do SUS, e que responderam ao inquérito nacional Qualiaids de 2016/2017.

O início do tratamento foi definido como a data registrada da primeira dispensação de medicamentos da TARV, enquanto o final do seguimento foi a data do óbito por doença do HIV ou da perda de seguimento (pessoa que, após 100 dias sem dispensação, não teve outra até o dia 31 de dezembro de 2018 nem registro de óbito por doença do HIV) ou a censura administrativa em 31 de dezembro de 2018.

A inclusão de pessoas de 15 anos ou mais ocorreu porque o protocolo clínico de tratamento não distingue adolescentes de adultos^11^. O período de acompanhamento considerou a aplicabilidade dos resultados do inquérito nacional dos serviços, realizado entre 2016 e 2017^10^.

### Fonte de Dados dos Indivíduos

No Brasil, o Ministério da Saúde (MS) consolida os registros dos serviços de saúde de assistência à infecção por HIV nos seguintes sistemas de informação: Sistema de Informação de Agravos de Notificação (Sinan), para notificar casos novos; Sistema de Informação sobre Mortalidade (SIM), para óbitos; Sistema de Controle Logístico de Medicamentos (Siclom), para dispensações de TARV de todas as pessoas em TARV residentes no país; e Sistema de Controle de Exames Laboratoriais (Siscel), de exames de contagem de linfócitos T-CD4 e de CV para as pessoas em acompanhamento nos serviços do SUS.

Como esses sistemas não têm campo identificador único, o MS os relaciona de modo probabilístico utilizando o programa Reclink^12^, validado para sistemas de HIV^13,14^. O banco relacionado constitui a base para os indicadores de saúde dos boletins epidemiológicos nacionais^15,16^ e já foi usado em estudos longitudinais de abrangência nacional^1,2,4^.

O recorte do banco relativo ao período de 2015 a 2018 com dados anonimizados e identificador único, obtido em 2020, foi a fonte primária dos dados dos indivíduos. Também foram acessados os bancos originais completos do Siclom e do Siscel para identificar inconsistências.

A análise de consistência objetivou identificar possíveis erros do relacionamento probabilístico (pessoas provavelmente diferentes que foram pareadas pelo programa e tiveram os registros originais reunidos em um único código identificador) e possíveis erros do registro original no preenchimento dos sistemas de informação por parte do serviço. Nessa etapa, foi realizado um relacionamento determinístico com os bancos do Siclom e Siscel para confirmar os pares verdadeiros e foram verificadas as distribuições das variáveis clínicas (dispensações de medicamento e exames de T-CD4 e CV) e feitas tabulações cruzadas para identificar erros.

Após exclusão das inconsistências, definiu-se o sistema de saúde de acompanhamento do tratamento do indivíduo: se em serviços ambulatoriais do SUS ou em serviços privados. Foram definidas como em acompanhamento no SUS as pessoas com: i) dois ou mais resultados de exames de CV; ou ii) apenas um resultado de exame de CV e perda de seguimento por abandono ou óbito em menos de 66 dias após a primeira dispensação de TARV. Esse intervalo se baseou no protocolo clínico da TARV em adultos, que recomenda a realização de exame de CV em até 56 dias após o início da TARV^11^. Por tratar-se de um estudo de “vida real”, acrescentaram-se dez dias ao intervalo recomendado, admitindo atrasos advindos do serviço e/ou do usuário.

O serviço de acompanhamento foi definido como aquele que solicita os exames de CV. Para as pessoas com apenas um serviço solicitante do exame de CV, a atribuição de serviço SUS foi direta; para aquelas com mais de um serviço solicitante de CV, o serviço atribuído foi aquele em que houve mais solicitações de CV ou no qual o acompanhamento foi mais longo.

Em seguida, foram selecionados os indivíduos com, no mínimo, duas CV e registro de primeiro exame de CD4 coletado em até 180 dias antes e 30 dias depois do início da TARV. Esse intervalo de tempo baseou-se na grande variação de intervalos observada no banco de dados original, frequente em estudos que utilizam dados de vigilância^17^. O procedimento resultou no banco de dados de características sociodemográficas e clínicas dos indivíduos (Figura 1).

**Figura 1:** Fluxograma do processo de construção da Coorte Qualiaids Brasil. Brasil, 2022.

Sinan: Sistema de Informação de Agravos de Notificação; Siclom: Sistema de Controle Logístico de Medicamentos; Siscel: Sistema de Controle de Exames Laboratoriais de CD4+/CD8+ e Carga Viral do HIV; SIM: Sistema de Informação sobre Mortalidade; PVHIV: pessoas vivendo com HIV; PVHA: pessoas vivendo com HIV/aids; TARV: terapia antirretroviral; CV: carga viral; ARV: antirretroviral.

^a^ Incompatibilidade dos dados do Sinan (n = 593), dispensação posterior ao óbito (n = 300), óbitos anteriores ao início de tratamento (n = 649), notificação de tuberculose posterior ao óbito (n = 67), resultados de exames de CD4 duplicados (n = 2), resultados de exames de CV duplicados (n = 261), registros de dispensação duplicados (n = 257), registro incorreto do início de TARV (5.073), interrupção de TARV e resultado posterior de CV indetectável (n =160).

^b^ Registro de primeiro resultado de CV indetectável (n = 19.081).

### Fonte de Dados dos Serviços

Foi utilizado o banco primário de respostas ao inquérito nacional Qualiaids de 2016/2017, respondido pela equipe técnica dos serviços ambulatoriais do SUS. O questionário Qualiaids é um instrumento de avaliação validado e periodicamente atualizado, já respondido em 2002, 2007 e 2010^6,18^. Ele contém perguntas sobre características gerais do serviço (tipo, número de pessoas em TARV e infraestrutura) e 60 questões que avaliam o desempenho, agrupadas nos domínios de acesso e prontidão, cuidado médico, cuidado multiprofissional, gerência e monitoramento da qualidade da assistência prestada e comunicação com usuários, população local, instituições da sociedade civil e rede local de saúde. A porcentagem de respostas positivas às questões avaliativas produz o escore de desempenho geral do serviço.

### Relacionamento entre o Banco dos Indivíduos e dos Serviços

Os bancos de dados individuais e dos serviços foram relacionados de modo determinístico por meio dos dados de localização do serviço presentes em ambos (Figura 1).

A base final da Coorte Qualiaids Brasil contém as variáveis dos indivíduos, sociodemográficas e clínicas, e as variáveis programáticas dos serviços de saúde que os acompanham (Quadro).

**Quadro:** Variáveis dos indivíduos e dos serviços incluídos na Coorte Qualiaids Brasil. Brasil, 2022.

Variável	Categorias/unidades	Base
**Indivíduo**		
Data de nascimento	dia:mês:ano	Banco relacionado
Sexo	masculino; feminino; ignorado	Banco relacionado
Raça/cor da pele	branca; preta; amarela; parda; indígena; ignorado	Banco relacionado
Escolaridade, em anos de estudo	não estudou; 1–3; 4–7; 8–11; ≥ 12; ignorada	Banco relacionado
Categoria de exposição	homossexual; heterossexual; bissexual; usuários de drogas injetáveis; hemofílico; transfusão; acidente material biológico; transmissão vertical; ignorado	Banco relacionado
Data do diagnóstico de tuberculose	dia:mês:ano	Banco relacionado
Data do encerramento do caso de tuberculose	dia:mês:ano	Banco relacionado
Datas das coletas de CD4	dia:mês:ano	Siscel
Resultados de CD4 (contagem de células LT- CD4)	mm^3^	Siscel
Data de registro da primeira dispensa	dia:mês:ano	Banco relacionado
Datas das retiradas de ARV	dia:mês:ano	Siclom
Datas das coletas de carga viral	dia:mês:ano	Siscel
Resultado da primeira carga viral	indetectável; detectável ou acima do limite de detecção	Siscel
**Serviço**		
Região metropolitana (RM) do serviço	RM brasileiras	Inquérito Qualiaids
Tipo de serviço do acompanhamento	ambulatório; atenção primária à saúde	Inquérito Qualiaids
Macrorregião do Brasil onde se situa o serviço	N; NE; SE; S; CO	Inquérito Qualiaids
Serviço localizado na capital	sim; não	Inquérito Qualiaids
Número de pessoas que iniciaram TARV sob acompanhamento no serviço	numérica	Siscel
Escore geral no questionário Qualiaids	numérica	Inquérito Qualiaids
Escore no domínio: acesso e prontidão	numérica	Inquérito Qualiaids
Escore no subdomínio: cuidado médico	numérica	Inquérito Qualiaids
Escore no subdomínio: cuidado multiprofissional	numérica	Inquérito Qualiaids
Escore no subdomínio: gerência e monitoramento da qualidade da assistência prestada	numérica	Inquérito Qualiaids
Escore no subdomínio: comunicação com usuários, população local, rede local de saúde, instituições da sociedade civil	numérica	Inquérito Qualiaids
Infraestrutura do serviço	suficiente; aceitável; insuficiente	Inquérito Qualiaids

Siclom: Sistema de Controle Logístico de Medicamentos; Siscel: Sistema de Controle de Exames Laboratoriais de CD4+/CD8+ e Carga Viral do HIV; TARV: terapia antirretroviral; ARV: antirretroviral; N: Norte; NE: Nordeste; SE: Sudeste; S: Sul; CO: Centro-Oeste.

### Análises Estatísticas

As variáveis individuais foram descritas segundo frequências absolutas e relativas. Apresentaram-se as frequências das características gerais do serviço segundo as macrorregiões brasileiras, enquanto os escores de desempenho dos serviços foram exibidos de acordo com domínio e macrorregião.

### Considerações Éticas

O projeto foi aprovado pelo Comitê de Ética em Pesquisas em Seres Humanos da Faculdade de Medicina da Universidade de São Paulo (USP) em 23 de janeiro de 2020 (CAAE 27659220.3.0000.0065; Parecer 3.807.435).

## RESULTADOS

A Coorte Qualiaids Brasil incluiu 132.540 pessoas vivendo com HIV/aids (PVHA), considerando os critérios de elegibilidade. O relacionamento dos bancos de dados secundários do SUS resultou em 273.150 observações, das quais 7.882 (2,9%) foram classificadas como inconsistentes e excluídas. Também foram identificados e excluídos 19.081 (7%) registros de pessoas com primeira carga viral com resultado indetectável (Figura 1).

Entre os participantes da coorte, 69,7% (92.361) eram do sexo masculino, 50,6% (67.004) pretos e pardos, 64,3% (85.206) com idade entre 20 e 39 anos e 54,8% (72.642) tinham de 4 a 11 anos de escolaridade. A categoria de transmissão foi heterossexual para 36% (47.657), mas ignorada para 34,1% (45.257) dos participantes. O CD4 mediano de base foi 419 células/mm^3^. Iniciaram o tratamento com CD4 menor de 200 células/mm^3^25,9% (34.398) dos participantes. Tiveram registro de pelo menos um episódio de tuberculose ativa 5,8% (7.747) dos indivíduos e 1,9% (2.515) foi a óbito por doença do HIV durante o período do estudo (Tabela 1).

**Tabela 1:** Perfil dos indivíduos incluídos na Coorte Qualiaids Brasil. Brasil, 2022 (n = 132.570).

Características	n	%
Sexo		
Masculino	92.361	69,7
Feminino	40.177	30,3
Ignorado	2	0
Raça/cor		
Branca	54.625	41,2
Pretos e pardos	67.004	50,6
Amarela	921	0,7
Indígena	258	0,2
Ignorado	9.732	7,3
Idade (em anos)		
15–19	5.962	4,5
20–39	85.206	64,3
40–49	23.961	18,1
≥ 50 anos	17.411	13,1
Escolaridade (em anos de estudo)		
0–3	11.024	8,3
4–11	72.642	54,8
≥ 12 anos	24.251	18,3
Ignorado	24.623	18,6
Categoria de exposição		
Homossexual/bissexual	37.397	28,2
Heterossexual	47.657	36,0
Usuário de drogas injetáveis	1.539	1,2
Outros	690	0,5
Ignorado	45.257	34,1
Registro de tuberculose ativa		
Sim	7.747	5,8
Óbito por doença do HIV		
Sim	2.515	1,9
Ano de início do tratamento do HIV		
2015	36.979	27,9
2016	36.649	27,7
2017	36.103	27,2
2018	22.809	17,2
Contagem inicial de linfócitos T-CD4 (células/mm^3^)		
< 200	34.398	25,9
200–349	25.718	19,4
350–499	27.140	20,5
> 499	45.284	34,2

Compuseram a Coorte Qualiaids Brasil 941 serviços de saúde do SUS, dos quais 59% (555) estão localizados na região Sudeste, 49% (461) concentravam de 51 a 500 participantes, 75,5% (710) foram caracterizadas como “tipo ambulatório” e 34,8% (327) apresentaram infraestrutura classificada como insuficiente (Figura 2).

**Figura 2:** Serviços do SUS de tratamento do HIV e número de pessoas incluídas na coorte que iniciaram TARV entre 2015 e 2018 segundo a macrorregião do país. Coorte Qualiaids Brasil. Brasil, 2022. (n = 941).

PVHA: pessoas vivendo com HIV/aids; TARV: terapia antirretroviral.

^a^ Os serviços foram classificados com base na resposta à questão sobre o tipo de serviço do questionário Qualiaids. “Tipo ambulatório” para as respostas: ambulatório exclusivo de assistência especializada a pacientes com HIV/aids, ambulatório de DST e hepatites virais; ambulatório especializado em moléstias infecciosas; equipe de assistência especializada inserida em um serviço de atenção básica; ambulatório de assistência a várias especialidades e ambulatório de hospital; “Tipo atenção básica” para as respostas: unidade básica de saúde, centro de saúde, saúde da família.

^b^ A infraestrutura dos serviços foi calculada com base nas respostas de 45 questões do questionário Qualiaids sobre a disponibilidade de: exames de avaliação inicial e seguimento, exames complementares para avaliação de condições clínicas adicionais frequentes dos pacientes com HIV/aids, encaminhamentos para médicos especialistas, modalidades assistenciais e demais profissionais de saúde, e disponibilidade de medicamentos e insumos para prevenção e diagnóstico. Os serviços que têm até 49% da infraestrutura esperada foram classificados como “insuficientes”, aqueles com infraestrutura entre 50% e 64% foram definidos como “aceitáveis” e os que dispõem de mais de 65% de infraestrutura disponível foram classificados como “suficientes”.

O desempenho médio da organização da assistência variou de 29% a 75% do padrão esperado. O domínio de gerência e monitoramento da qualidade da assistência prestada apresentou o pior escore (Tabela 2).

**Tabela 2:** Desempenho dos serviços ambulatoriais do SUS que acompanham pessoas que iniciaram o tratamento do HIV entre 2015 e 2018 segundo proporção média de respostas positivas ao inquérito do questionário Qualiaids 2016/2017. Coorte Qualiaids Brasil. Brasil, 2022. (n=941).

Domínios avaliados^a^	Centro-Oeste			Nordeste			Norte			Sudeste			Sul			Brasil		
					
	escore	med	DP	escore	med	DP	escore	med	DP	escore	med	DP	escore	med	DP	escore	med	DP
Acesso e prontidão	0,53	0,5	0,2	0,5	0,5	0,21	0,55	0,5	0,18	0,61	0,63	0,23	0,53	0,5	0,2	0,58	0,63	0,22
Cuidado médico	0,56	0,6	0,17	0,57	0,6	0,21	0,56	0,6	0,2	0,67	0,7	0,21	0,6	0,6	0,19	0,63	0,6	0,21
Cuidado multiprofissional	0,76	0,79	0,19	0,72	0,71	0,26	0,76	0,86	0,23	0,76	0,86	0,24	0,71	0,79	0,26	0,75	0,86	0,24
Gerência e monitoramento da qualidade da assistência prestada	0,33	0,3	0,2	0,34	0,3	0,16	0,29	0,3	0,18	0,35	0,3	0,17	0,32	0,3	0,16	0,34	0,3	0,17
Comunicação com usuários, população local, rede local de saúde, instituições da sociedade civil	0,22	0,14	0,16	0,26	0,29	0,19	0,26	0,29	0,19	0,31	0,29	0,2	0,26	0,29	0,19	0,29	0,29	0,2
Total	0,5	0,48	0,1	0,49	0,52	0,15	0,5	0,5	0,13	0,57	0,58	0,15	0,51	0,52	0,13	0,54	0,55	0,15

med: média; DP: desvio-padrão.

^a^ As 60 questões de avaliação do questionário Qualiaids estão agrupadas em cinco domínios, com a seguinte distribuição: 16 questões sobre atividades realizadas no primeiro atendimento, disponibilidade de teste para HIV, profilaxias pré e pós-exposição e tipos de atendimentos realizados pelo serviço compõem o domínio de “acesso e prontidão”; 20 questões sobre as atividades realizadas pelo médico compõem o domínio “cuidado médico”; 7 questões sobre as atividades realizadas pelo enfermeiro, farmacêutico, psicólogo, assistente social e técnico de enfermagem constituem o domínio de “cuidado multiprofissional”; 10 questões sobre realização de reuniões, monitoramentos e registros formam o domínio de “gerência e monitoramento da qualidade da assistência prestada”; e 8 questões sobre tipos e formas de comunicação do serviço compõem o domínio “comunicação com usuários, população local, rede local de saúde, instituições da sociedade civil”.

## DISCUSSÃO

Foi possível construir uma coorte que, de modo inédito no Brasil, integra dados disponíveis, mas antes não relacionados, das pessoas em tratamento do HIV e dos serviços de saúde do SUS que as assistem.

Dados provenientes de bases administrativas têm sido utilizados em diversos estudos sobre HIV^21^, porém exigem detalhada análise de consistência, ainda mais crucial em um estudo como este, que combina dados de um inquérito com aqueles provenientes de um banco de dados previamente relacionado. As informações de características sociodemográficas e clínicas provêm do relacionamento probabilístico conduzido anualmente pelo MS, utilizado em estudos publicados em revistas com seletiva política de revisão por pares^1,2,4^. Contudo, foram realizadas avaliações adicionais de potenciais erros de atribuição de código identificador único ou de registro, por comparação com os principais bancos originais.

Encontraram-se 19.081 (7%) registros de pessoas com resultado da primeira CV indetectável. Destes, foram identificados no histórico de CV 520 registros que parecem ser de pessoas que mantêm CV indetectável por longo tempo (“controladores de elite”), cuja prevalência estimada é menor do que 1%^1,24^. Assim, é provável que a maioria dos casos resulte do chamado “registro tardio” no Siclom, isto é, pessoas que iniciaram tratamento, receberam medicação, mas não foram registradas imediatamente no sistema. Erros como esse refletem irregularidade da qualidade dos registros entre os serviços. Já os erros cuja origem mais provável foi o relacionamento probabilístico ocorreram em menor proporção (2,7%). Todos os erros foram objeto de relato detalhado para a gestão federal de HIV do MS, responsável pelo gerenciamento dos sistemas e pela execução do relacionamento. Por fim, ressalta-se que o relacionamento dos sistemas em nível nacional, por meio de um código identificador único, seria o cenário ideal. Enquanto não há essa condição, os relacionamentos probabilísticos seguem como fontes valiosas para estudos de vida real^23^.

A inclusão somente de pessoas que fazem seu acompanhamento clínico laboratorial no SUS, necessidade ditada pelo desenho do estudo, implicou a exclusão de 30% dos indivíduos. Entre eles, 63% são pessoas que utilizam os serviços do SUS apenas para dispensação de medicamentos prescritos por serviços privados, assim, não são obrigados a fornecer informações clínicas para os sistemas de informação do SUS. Essa exclusão não representa uma limitação, uma vez que decorre de característica inerente à organização da atenção em saúde do Brasil. Representa, ao contrário, uma fortaleza, já que este é o primeiro estudo nacional brasileiro que ajusta a inclusão segundo tal característica. Adicionalmente, a estimativa inédita sobre o papel relativo dos sistemas público e privado na assistência em HIV no Brasil compôs produto já publicado deste estudo^25^.

Entre os indivíduos acompanhados por serviços do SUS, a ausência de registros de CV e/ou CD4 implicou exclusão de 8%. Embora o monitoramento laboratorial oportuno seja recomendado no protocolo clínico de tratamento^11^, a realização depende, sobretudo, da correta observância por parte do médico assistente. A insuficiência do monitoramento laboratorial já foi apontada no Brasil^26^ e na América Latina^27^. Já a não resposta dos serviços ao inquérito excluiu apenas 3%.

O perfil sociodemográfico e clínico dos participantes assemelha-se ao observado em coorte nacional anterior^1,2^. O perfil dos serviços confirma a heterogeneidade estrutural e de desempenho apontada em estudos anteriores^6^. Ressalta a insuficiência da gerência e monitoramento da qualidade, que afeta particularmente a identificação e intervenção de pessoas em falha terapêutica e/ou abandono de tratamento^28,29^ e tem papel crucial para aprimorar o desempenho geral do serviço de assistência^30^.

O relacionamento entre bases de dados individuais e dos serviços possibilita, pela primeira vez no país, o diálogo com estudos internacionais de desenho semelhante^8,33^. Espera-se, assim, que a Coorte Qualiaids Brasil contribua para a construção de evidências na área, a partir de análises que integrem os diferentes níveis envolvidos na determinação de resultados do tratamento do HIV de pessoas acompanhadas nos serviços do SUS.
